# Spread of Epidemic Disease on Edge-Weighted Graphs from a Database: A Case Study of COVID-19

**DOI:** 10.3390/ijerph18094432

**Published:** 2021-04-22

**Authors:** Ronald Manríquez, Camilo Guerrero-Nancuante, Felipe Martínez, Carla Taramasco

**Affiliations:** 1 Laboratorio de Investigación Lab[e]saM, Departamento de Matemática y Estadística, Universidad de Playa Ancha, 2340000 Valparaíso, Chile; 2 Escuela de Enfermería, Universidad de Valparaíso, 2520000 Viña del Mar, Chile; camilo.guerrero@uv.cl; 3 Facultad de Medicina, Escuela de Medicina, Universidad Andrés Bello, 2520000 Viña del Mar, Chile; felipe.martinez.l@unab.cl; 4 Escuela de Ingeniería Civil Informática, Universidad de Valparaíso, 2340000 Valparaíso, Chile; carla.taramasco@uv.cl; 5 Centro Nacional de Sistemas de Información en Salud, 8320000 Santiago, Chile

**Keywords:** edge-weighted graph, SIR model, network, disease, COVID-19

## Abstract

The understanding of infectious diseases is a priority in the field of public health. This has generated the inclusion of several disciplines and tools that allow for analyzing the dissemination of infectious diseases. The aim of this manuscript is to model the spreading of a disease in a population that is registered in a database. From this database, we obtain an edge-weighted graph. The spreading was modeled with the classic SIR model. The model proposed with edge-weighted graph allows for identifying the most important variables in the dissemination of epidemics. Moreover, a deterministic approximation is provided. With database COVID-19 from a city in Chile, we analyzed our model with relationship variables between people. We obtained a graph with 3866 vertices and 6,841,470 edges. We fitted the curve of the real data and we have done some simulations on the obtained graph. Our model is adjusted to the spread of the disease. The model proposed with edge-weighted graph allows for identifying the most important variables in the dissemination of epidemics, in this case with real data of COVID-19. This valuable information allows us to also include/understand the networks of dissemination of epidemics diseases as well as the implementation of preventive measures of public health. These findings are important in COVID-19’s pandemic context.

## 1. Introduction

Infectious diseases have been the object of study throughout the history of mankind. Multiple disciplines have contributed to the understanding of these health phenomena, in particular the sources and types of infections, as well as the negative consequences on the population.

From an epidemiological and health perspective, humanity has experienced a series of infectious disease events, including Cholera, Malaria, and AIDS [1]. Infectious diseases have an epidemic potential due to the dissemination of microorganisms, generally viruses that develop in a host and later seek another living being to continue with their survival process [2,3]. Therefore, the spread of this type of disease occurs through contact between living beings, humans or animals, which present significant loads of pathogenic microorganisms. Consequently, when massive infections occur, we are facing an epidemic outbreak. The concept of an epidemic is established when the infectious outbreak affects a specific geographic area and a pandemic is related to an event spread over extensive continental areas [4].

An example of the above is the current COVID-19 pandemic context, the study of the spread of diseases being of interest [5,6]. The beginning of the pandemic was registered in the city of Wuhan, China [7]. The consequences of the COVID-19 pandemic have been evidenced in a series of dimensions, including the collapse of health systems in some countries, the stoppage of production, the impoverishment of communities, unemployment, among other social and economical consequences [8].

In this sense, the current and historical contributions of mathematical models are important. The compartmental models are useful to establish in a simple way the projections and evolution of infectious diseases. They are characterized by compartmentalizing the population depending on whether the disease generates immunity or not [3]. One of the classic compartmental mathematical models is SIR, developed by Kermack and Mc Kendrick in 1927 for the understanding of epidemics [9], and the current use of computational simulations is of great relevance to analyze the behavior, in this case, of SARS-CoV2 (see, for instance, [10]). The SIR model compartmentalizes or divides the population into Susceptible (S), Infected (I), and Removed (R). This compartmentalization allows for analyzing the population with these states and is useful to determine projections in relation to the total number of patients and the duration of the disease [11]. The SIR model approach is eminently deterministic; however, it has also been used from a stochastic perspective, improving the representation of the dynamics of infectious diseases through the probability of the appearance of epidemic outbreaks [11]. There are other mathematical models in epidemiology that have been developed from the SIR model, adding variables such as exposure and the effect of quarantine measures such as the SEIR and SEQIJR model, respectively [12]. With this, nations and governments can count on information to establish mitigation measures for the consequences of the virus, such as: safeguarding employment, strengthening health system responses, developing community actions, among other measures.

However, these models are limited when the extension and heterogeneity of the data are wide, so they fail to detect changes in the population structure and the variation in contact dynamics over time [12].

**Figure 1:**
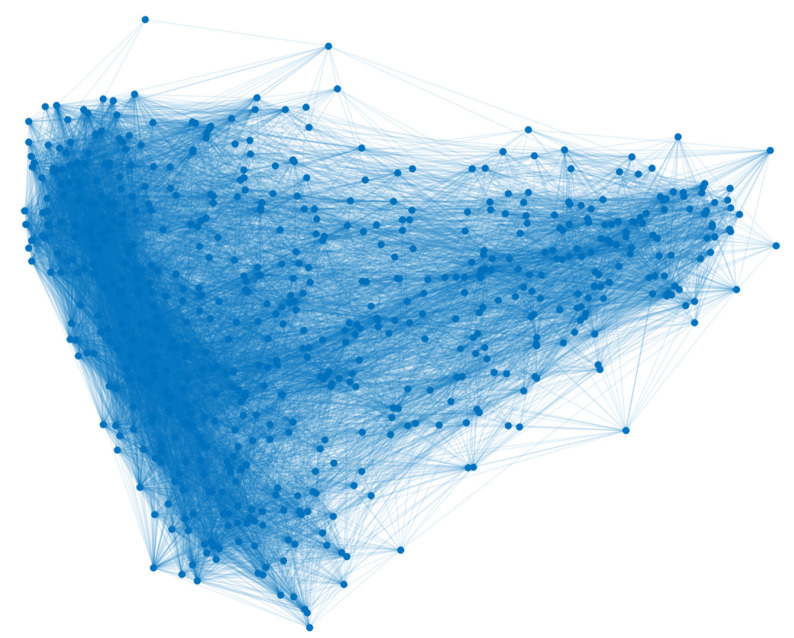
Network model of brain functional coactivations.

On the other hand, globalization and high population concentration have led to the inclusion of other ways of representing the spread of infectious diseases. Models with stochastic approximations have the advantage of establishing probabilities of person-to-person contact [3]. One of them is the network model, which is based on the theory of graphs studied from the observations of Leonhard Euler with the problem of the seven bridges. The model proposes the formation of individuals (nodes) and their relationship with others (edge), so the result is a network [12] (see, for instance, Figure 1).

To build a network model, the variables that are relevant to the spread of a disease are established. Among the multiple models developed, sociocentric studies stand out, which allow a broad exploration of the complete network that is generated to understand the spread of a pathogen. Therefore, network models are useful to understand the development of different infectious diseases [13]. It is not new, especially if the spread of an epidemic disease in network structures is studied, which in an abstract way is the main object of graph theory; see, for instance, in [14], where the authors use the model as a predictive tool, to emulate the dynamics of Ebola virus disease in Liberia, and in [15] where the transmission connectivity networks of people infected with highly contagious Middle East respiratory syndrome coronavirus (MERS-CoV) in Saudi Arabia were assessed to identify super-spreading events among the infected patients between 2012 and 2016. The relevance of these studies is related to the possibility of preventing nodes (people) from continuing to infect, an issue that is treated in [16] with the graph protection methods proposed by Wijayanto and Murata. Deepening in this line of studies, there have been included new mathematical models with variables related to the behavior of people associated with information and emotions during epidemics [17]. Likewise, dynamic models have covered the influence of the infectious disease itself on the network of contacts and, therefore, changes in the dynamics of spread of the epidemic over time [12].

In general, network models can be analyzed through static graphics such as snapshots. However, for an adequate approximation and correcting the loss of data generated by the snapshot, data modeling techniques must be used including the weighting of the edges and, consequently, better estimated, given the information obtained from the relationships between individuals and the spreading of the disease [12].

In the current context, organized information has an important value for the management of epidemics. The databases elaborated from the information of individuals can contribute in the characterization and knowledge of a determined area, which are important in order to know the evolution of the diseases [18].

In the case of infectious pathologies, depending on the type of database, it is possible to determine through the variables whether two or more individuals are linked to each other, such as people who live in the same neighborhood or work in the same place. Given the characteristics of network models and the obtaining of information through complex databases, it is relevant to use these models, in particular, with approximations that incorporate weighting on the edges.

For all the above, there are challenges around the possibility of representing and understanding the evolution of infectious diseases through a network model using databases. The relevance of this type of research contribution is based on the possibility of having tools that favor measures to prevent the spread of this type of disease, an issue that takes on greater social and scientific value due to the context of the SARS-CoV2 pandemic. Consequently, the aim of this article is to develop a spreading stochastic model of some disease from a database, particularly using variables that link individuals in a given territory, the probability of contagion among them, and, therefore, the spread of the disease through edge-weighted graphs (or edge-weighted networks). For the purposes of this manuscript, a database is understood as a matrix whose columns are the variables, while the rows correspond to the responses of the subjects in relation to the variables consulted.

Our proposal provides an edge-weighted graph obtained from a database that contains enough information about the individuals that belong to a population. With this graph, we use a graph-based SIR model in which each individual is represented by a vertex in an edge-weighted graph. At time *t*, each vertex $v_{i}$ is in a state $v_{i}^{t}$ belonging to S={0,1,−1}, where 0,1 and −1 represent the three discrete states: susceptible (S), infected (I), and recovered (R). We choose predetermined values for the parameters of the model changing only one of them at a time, observing the effects on the epidemic. These parameters are: the order of the graph, the mean degree of its vertices, the representative factor of the disease, the numbers of relation variables of database, and the numbers of classes (for the last parameters, see Section 3). The initial population contains one infected individual and all the simulations were done on a random and scale-free graph. The recovery rate is fixed in $\delta =\frac{1}{15}$. We have studied the effects of these parameters on two characteristics of the disease: the number of total infected and the duration of the disease. Finally, we tested our model with real COVID-19 data from Olmué City (Chili). The database was obtained from the epidemiological surveillance system of the Chilean Ministry of Health.

The paper is organized as follows: Section 2 contains generalities about graph theory and the SIR model. Section 3 is divided into two parts: first, we give the construction of the graph from a database. In the second part, we describe the Graph-based SIR model. In Section 4, we ran the different simulations of the spread of a disease on two different types of graphs and analyzed the effects of each parameter on the end of the disease and the number of infected. In Section 5, we give a deterministic approach to the Graph-based SIR model. In Section 6, we tested our model with database COVID-19 from Olmué City (Chili). Finally, in Section 7, we provide a discussion about the results of the Section 4, Section 5 and Section 6.

## 2. Basic Definitions

### 2.1. Graph

The following definitions come from [19,20].

**Definition** **1.** *A graph G is a finite nonempty set V of objects called vertices together with a possibly empty set E of 2-element subsets of V called edges.*

To indicate that a graph *G* has vertex set *V* and edge set *E*, we write G=(V,E). To emphasize that *V* and *E* are the vertex set and edge set of a graph *G*, we often write *V* as V(G) and *E* as E(G). Each edge {u,v} of *G* is usually denoted by uv or vu. If e=uv is an edge of *G*, then *e* is said to join *u* and *v*.

If uv is an edge of *G*, then *u* and *v* are adjacent vertices. Two adjacent vertices are referred to as neighbors of each other. The set of neighbors of a vertex *v* is called the open neighborhood of *v* (or simply the neighborhood of *v*) and is denoted by N(v). If uv and vw are distinct edges in *G*, then uv and vw are adjacent edges.

**Definition** **2.** *The number of vertices in a graph G is the order of G and the number of edges is the size of G.*

**Definition** **3.** *The degree of a vertex v in a graph G, denoted by $d(v_{i})$, is the number of vertices in G that are adjacent to v. Thus, the degree of v is the number of vertices in its neighborhood N(v).*

**Definition** **4.** *Let G be a graph of order n, where $V\left(G\right)=\left\{v_{1},v_{2},\;\ldots ,\;v_{n}\right\}$. The adjacency matrix of G is the n×n zero-one matrix $A\left(G\right)=\left[a_{ij}\right]$, or simply $A=[a_{ij}]$, where*

$$a_{ij}=\left\{\begin{array}{cc} 1 & if\;\;v_{i}v_{j}\in E\left(G\right)\\ 0 & if\;\;v_{i}v_{j}\notin E\left(G\right). \end{array}\right.$$

**Remark** **1.** *If we add the entries in row i (or in column i), then we obtain the degree of $v_{i}$.*

On the other hand, an important generalization is that the simple graph consists of the definition of weighted graph, more specifically edge-weighted graph. Informally, an edge-weighted graph is a graph whose edges have been assigned weights.

**Definition** **5.** *An edge-weighted graph a pair (G,W), where G=(V,E) is a graph and W:E→R is a weight function. If $v_{i}v_{j}\in E$, then $W\left(v_{i}v_{j}\right)=w_{ij}$.*

**Definition** **6.** *The strength of a vertex $v_{i}$, denoted $S(v_{i})$, is defined as the sum of the weights of all edges incident to it, this is to say*

$$S\left(v_{i}\right)=\sum_{v_{j}\in N\left(v_{i}\right)}w_{ij}.$$

### 2.2. Graph Classes and Basic Network Models

In this work, random and scale-free graph are used.

The first theoretical model of random networks is the classical random graph model, the most famous one being the Erd$\ddot{o}$s and Rényi in [21]. On the other hand, the scale-free graphs (or networks) are graphs whose distribution degree follows a power law distribution with an exponent between 2 and 3. This paper uses the model proposed by Albert-László Barabási and Réka Albert in [22].

### 2.3. SIR Model

In the entire spectrum of epidemiological models that currently exist, the SIR model is the basis or the simplest of all these.

The classical Kermack–McKendrick SIR model [9], developed in the early 1900s (see [23,24]), consists of a system of nonlinear ordinary differential equations, which expresses the spread among the population of a constant size, denoted by *N*, for all time *t*. The population is divided into three groups: susceptible individuals, infected individuals, and recovered (or removed) individuals. The sizes of these groups at time *t* are denoted by S(t), I(t), and R(t), respectively, such that N=S(t)+I(t)+R(t). The model is the following:

(1) $$\begin{array}{cc} \dot{S}\left(t\right) & =-\beta S(t)I(t) \end{array}$$

(2) $$\begin{array}{cc} \dot{I}\left(t\right) & =\beta S(t)I(t)-\delta I(t) \end{array}$$

(3) $$\begin{array}{cc} \dot{R}\left(t\right) & =-\delta I(t), \end{array}$$

t∈[0,T], subject to the initial conditions $S\left(0\right)=S_{0}$, $I\left(0\right)=I_{0}$ and $R\left(0\right)=R_{0}$. where the disease transmission rate β>0 and the recovery rate δ>0 (the duration of infection $\delta^{-1}$).

In summary, the above system describes the relationship between the three groups, this is to say, a susceptible individual changes its state to infected with probability β, while an infected changes its state to recovered with probability δ.

## 3. Model Description

This section is divided into two parts. A part dedicated to build a graph from a database and the second one to describe the dynamics of the disease on the graph-based SIR model.

To begin, we need some basic elements to understand what follows. First, we will understand by *variable* the characteristic assigned to a person from a predetermined set of values which can be a numerical measure, a category or a classification—for instance, income, age, weight, occupation, address, etc. Second, we will understand by *database* a matrix whose columns are variables, while the rows correspond to the responses of the subjects in relation to the variables consulted.

### 3.1. Graph from a Database

Let us consider a database, denoted by D, that stores information on *N* individuals of a population. Let *V* be the set of the persons registered in the database, equivalent

$$V=\left\{v_{1},v_{2},\;\ldots ,\;v_{N}\right\},$$

where $v_{i}$ is a person registered in D for i=1,…,N.

Let $v_{i}$ be a person registered in D for i=1,…,N. We set

$$EPI=\left\{X_{1},X_{2},\;\ldots ,\;X_{K}\right\},$$

where *K* is the number of elements of the set EPI. (*K* is the number of variables in D), $X_{k}$ is a variable in D for k=1,…,K, and EPI(i,k) is the response of the person $v_{i}$ to the variable $X_{k}$.

As we want to study the link between the people who are registered in D through the variables of this database, we must identify which are the variables that allow us to establish these links that promote the spread of the disease. For example, if two people are the same age, they do not necessarily meet and spread the virus unlike two people who live in the same city.

**Definition** **7.** *We will say that X∈EPI is a relationship variable if and only if it allows us to assume that some person meets another. In another case, we will say that X is a characteristic variable.*

The above allows us to define the following sets:

$$REL=\left\{X\in EPI:X\;\mathrm{is}\;a\;\mathrm{relationship}\;\mathrm{variable}\right\}$$

$$CHAR=\left\{X\in EPI:X\;\mathrm{is}\;a\;\mathrm{characteristic}\;\mathrm{variable}\right\}.$$

Let us denote by $K_{1}$ and $K_{2}$ the cardinality of REL and CHAR, respectively. Notice that $K_{1}+K_{2}=K$.

**Definition** **8.** *We will say that a person $v_{i}$ is related to a person $v_{j}$ if and only if there exists $X_{k}\in REL$ for $k\in \{1,2,\;\ldots ,\;K_{1}\}$ such that EPI(i,k)=EPI(j,k) and i≠j.*

It is clear that, if $v_{i}$ is related to $v_{j}$, then $v_{j}$ is related to $v_{i}$, this is to say that the relation is a symmetric relation.

The previous definition allows us to construct a graph *G* of links given by the relationship variables of the D. *G* will be considered as an undirected graph without loops or multiple edges.

On the other hand, it is possible that $k\in \left\{1,\;\ldots ,\;K_{1}\right\}$ is not unique because more than one variable may coincide. This induces us to define the weight of the link between $v_{i}$ and $v_{j}$.

### 3.2. Weighting Variables

In order to define the weight of each link between two vertices, we assume that each X∈REL has an associated inherent weight; this is to say, it is possible to discriminate some hierarchical order between the variables. Let $p_{k}$ be the weight associated with the variable $X_{k}\in REL$ for $k=1,\;\ldots ,\;K_{1}$.

On the other hand, to better understand the definition that follows and its consequences, suppose that *X* is a set of 100 people. If we define the relationship in the set: *person Q is related to person W if and only if they are the same age*, then we could group the people in the set by age. In addition, an interesting fact is that thanks to this relationship everyone would be part of a group and no one could be in two groups at the same time. These types of relations defined on a set are called *equivalence relations* and each defined group is called *equivalence class*.

**Definition** **9.** *We will say that, for $X_{j},X_{t}\in REL$, $X_{j}$ is related to $X_{t}$, denoted by $X_{j}RX_{t}$, if and only if $p_{j}=p_{t}$.*

**Lemma** **1.** *The relation R defined on REL is an equivalence relation.*

**Proof.**  Directly. □

Thanks to the relation R, we can consider the different equivalence classes which are composed of the variables that have the same weight. Hence, we have the same number of classes as different weights.

**Definition** **10.** *Let $A_{1},A_{2},\;\ldots ,\;A_{c}$ be the different classes that are defined by the different weights $p_{1},p_{2},\ldots ,p_{c}$ and $\alpha_{1},\alpha_{2},\;\ldots ,\;\alpha_{c}$ its respective cardinalities. Hence,*

(4) $$p_{j}=\frac{\alpha_{j}}{K_{1}},$$

*for all j∈{1,2,…,c}.*

### 3.3. Weighted Link

The aim in this subsection is to introduce the definition of weight link.

Let $v_{i},v_{j}\in V$ such that $v_{i}$ is related to $v_{j}$. We set

(5) $$\begin{array}{cc} H & =\left\{k\in \left\{1,\;\ldots ,\;K_{1}\right\}:EPI\left(i,k\right)=EPI\left(j,k\right)\right\}. \end{array}$$

We denoted by $h_{i,j}$ the cardinality of the set *H*. Notice that $h_{i,j}$ is simply the number of times that one person is related to another (or the number of variables that matches between them). Since our proposal considers undirected graph, we have that $h_{i,j}=h_{j,i}$.

**Definition** **11.** *Let $v_{i},v_{j}\in V$ be such that $v_{i}$ is related to $v_{j}$ and $p_{k_{r}}$ the weight of the variable in which $v_{i}$ and $v_{j}$ match, for $r=1,\;\ldots ,\;h_{i,j}$. We will say that*

(6) $${\tilde{w}}_{ij}=\sum_{r=1}^{h_{i,j}}p_{k_{r}},$$

*is the weight of the link between $v_{i}$ and $v_{j}$.*

Finally, the weighted adjacency matrix of *G* is the n×n matrix $A\left(G\right)=\left[a_{ij}\right]$, where

$$a_{ij}=\left\{\begin{array}{cc} {\tilde{w}}_{ij} & \mathrm{if}\;\;v_{i}v_{j}\in E\left(G\right)\\ 0 & \mathrm{if}\;\;v_{i}v_{j}\notin E\left(G\right). \end{array}\right.$$

The idea of having a graph with weights in its edges is to be able to differentiate or measure, in some way, the strength or closeness between individuals. For example, it is not the same saying that two individuals share the same city than saying they share the same house they live in; it follows that the latter makes the relationship closer and consequently the contagion of the disease is intuitively more likely.

**Example** **1.** *In the following example, Table 1 simulates a database with 20 registered people. The data hosted correspond to the city in which they live (City), workplace (considering school and university as a workplace), gender (Gen.), age, extracurricular activity (EC activity), address, if they drink alcohol (Drin.), if they are smokers (Sm.), and marital status (MS). Let us consider A and B two different cities, x,y,z,w,u,v,r,s,q,t,p,k,d,g and h different people’s addresses. Moreover, in the table, Y = Yes, N = No, IC = in couple, M = married, S = single, W = widower.*

*From Table 1, we have that $EPI=\{X_{1},X_{2},X_{3},X_{4},X_{5},X_{6},X_{7},X_{8},X_{9}\}$, where $X_{1}$ = City, $X_{2}$ = Workplace, $X_{3}$ = E.P. activity, $X_{4}$ = Address, $X_{5}$ = Sm., $X_{6}$ = Dri., $X_{7}$ = Gen., $X_{8}$ = M.S. and $X_{9}$ = Age. Then, by Definition 7, we obtain the sets:*

*1*  *$REL=\{X_{1},X_{2},X_{3},X_{4}\}$ and*
*2*  *$CHAR=\{X_{5},X_{6},X_{7},X_{8},X_{9}\}$.*

*In our criteria, the hierarchical order of the variables $X_{1},X_{2},X_{3},X_{4}$ in descending form is $X_{4},X_{2},X_{3}$, and $X_{1}$. Moreover, we consider that the variables $X_{4}$ and $X_{2}$ have the same weight. Hence, $A_{1}=\left\{X_{2},X_{4}\right\}$, $A_{2}=\left\{X_{3}\right\}$, and $A_{3}=\left\{X_{1}\right\}$ are the different classes that are defined by the different weights. Hence, by Definition 10*

$$p_{1}=\frac{1}{2},\;p_{2}=\frac{1}{4},\;p_{3}=\frac{1}{4}.$$

**Figure 2:**
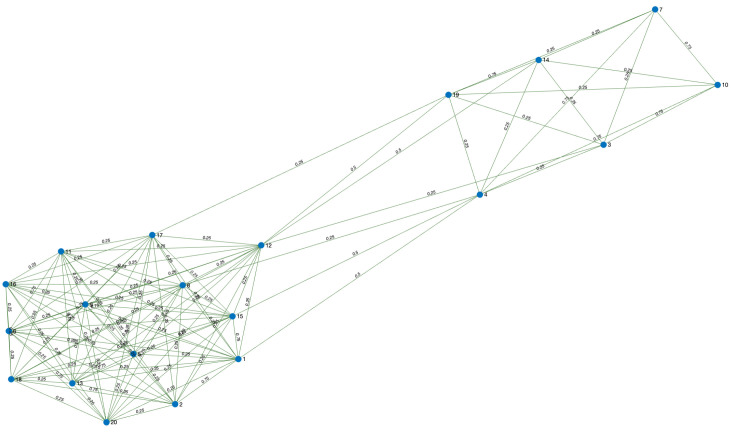
Graph obtained from D.

*To construct the graph, we must resort to Definition 8. For instance, person 17 is related to all the people who live in city A or who work at Workplace 8 or who have music as an extra curricular activity or whose address is k. With respect to the weights of the edges, Equation (6) in Definition 11 gives us the answer. For instance, person 6 matches person 11 in the answers of the variables $X_{1}$ and $X_{2}$, this is to say, both people live in city A and have the same workplace. Then, the edge $v_{6}v_{11}$ has weight $w_{6\;11}=0.5+0.25=0.75$. Figure 2 shows the obtained graph.*

### 3.4. Graph-Based SIR Model

Having described a population with a network model, the spreading of an epidemic is modeled by a dynamic system that uses the graph (in our case an edge-weighted graph) as its support. The class of chosen model is the probabilistic cellular automata (see [25]), this is to say, the model in which the events happen at times t=0,Δt,2Δt,…, where Δt is the discretization interval. In this work, we use a graph-based SIR model in which each individual is represented by a vertex in an edge-weighted graph. At time *t*, each vertex $v_{i}$ is in a state $v_{i}^{t}$ belonging to S={0,1,−1}, where 0,1 and −1 represent the three discrete states: susceptible (S), infected (I), and recovered (R).

Let *G* be the edge-weighted graph obtained from a database D and $v_{i}\in V\left(G\right)$. We set

(7) $$N_{I}\left(v_{i}\right)=\left\{v\in N(v_{i}):v\in I\right\}.$$

At time t+Δt, the vertex $v_{i}$ will change state according to probabilistic rules:

1 The probability ($P_{I}\left(v_{i}\right)$) that a susceptible vertex $v_{i}$ is infected by one of its neighbors is given by
  (8) $$P_{I}\left(v_{i}\right)=\sum_{v_{j}\in N_{I}\left(v_{i}\right)}\rho \Delta t\cdot {\tilde{w}}_{ij},$$
  where ρ is a purely biological factor and representative of the disease.
2 The probability ($P_{R}\left(v_{i}\right)$) that a infected vertex $v_{i}$ at time *t* will recover is given by
  (9) $$P_{R}\left(v_{i}\right)=\delta \Delta t,$$
  where δ is the recovery rate.

Moreover, we assume that the disease is present for a certain period of time and, when individuals recover, they are immune.

**Remark** **2.** *Notice that the expression (8) can be deduced from the infection model called q-influence, assuming q=ρ. (see [26,27]).*

## 4. Simulation of Disease Spread

In this section, we choose predetermined values for the parameters changing only one of them at a time, observing the effects on the epidemic. For a given type of graph, these parameters are: the order of the graph (n=1000), the mean degree of its vertices (m=6), the representative factor of the disease (ρ=0.015), the amount of relation variables of database ($K_{1}=20$), and the amount of classes (c=6). The initial population contains one infected individual and all the simulations were done on a random and scale-free graph and considering $\delta =\frac{1}{15}$. Moreover, the software in which the simulations were run correspond to Matlab in its R2020b version. Finally, the source code of our analysis and network files are accessible through the Github link: https://github.com/RonaldManriquez/Spread-of-disease.git (accessed on 20 February 2021).

We want to study the effects of these parameters on two characteristics of the disease: the number of total infected and the duration of the disease. We have reparameterized the number of total infected $\left(\frac{I(T)}{N}\right)$ while the duration of the disease is simply the end of it, this is to say, when the infected are 0.

**Figure 3:**
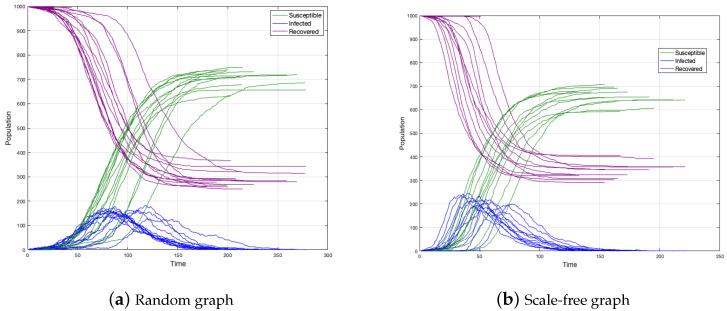
Epidemic spreading on random and scale-free graph simulation.

We simulated 30 epidemic spreadings on two graphs: a random graph and a scale-free graph to see how epidemics differ from each other (see Figure 3a,b).

In both cases, the number of total infected is between 600 and 750 individuals and the duration of the disease around 150–200 time units. We concluded then that epidemics do not differ from each other. In a simple analysis, the peak in the scale-free graph occurs before the case of the random graph. Perhaps the above is because, in the scale-free graph, the disease spreading is faster.

### 4.1. Order of the Graph

**Figure 4:**
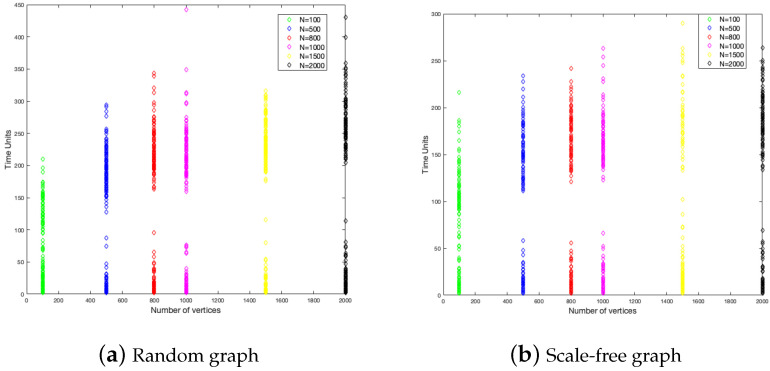
Duration of the disease in random and scale-free graph.

**Figure 5:**
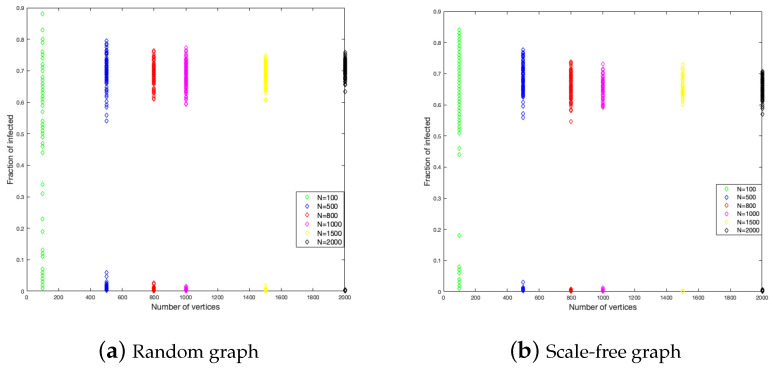
Fraction infected in random and scale-free graph.

To study the graph size influence on the duration of the disease and the fraction of infected, we have considered six different sizes of graphs, these are 100, 500, 800, 1000, 1500, and 2000. In each one of them, we did 200 simulations. The results are shown in Figure 4 and Figure 5.

Figure 4 shows that the end of the disease is farther for the random graph than the scale-free graph. The random graph has around 350 time units as a maximum, while the scale-free graph has around 250 time units. This is to say, in the last case, the disease epidemic is shorter. This is a constant for each size of the graph.

With respect to the fraction of the infected, the random graph is more homogeneous in the numbers of individuals that get infected with a little influence from the size, while, in the scale-free graph, the fraction of infected is decreasing when the size is increasing.

### 4.2. Representative Factor of the Disease (ρ)

**Figure 6:**
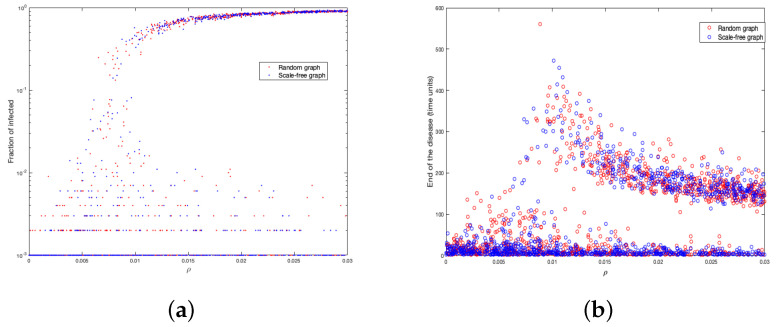
Fraction of infected for different values of ρ (**a**); end of the disease for different values of ρ (**b**).

In this subsection, we study how the representative factor of the disease (ρ) affects the two epidemic parameters we are interested in. We did 1000 simulations with values of ρ randomly sampled from a uniform distribution between 0 and 0.03. In Figure 6a, we show the change of the fraction of infected in function of ρ and in Figure 6b the variation of end of the disease according to ρ.

Notice that, from ρ=0.015, the fraction of infected tends more clearly to 1, while near ρ=0.005, close to 1% of the population is infected. On the other hand, when ρ is close to 0.01, the disease has a longer duration (very close to the value by settings).

### 4.3. The Mean Degree of Vertices

**Figure 7:**
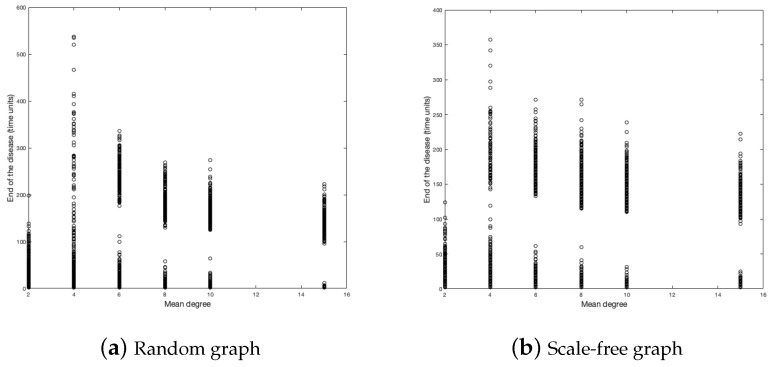
Duration of the disease in random and scale-free graph.

**Figure 8:**
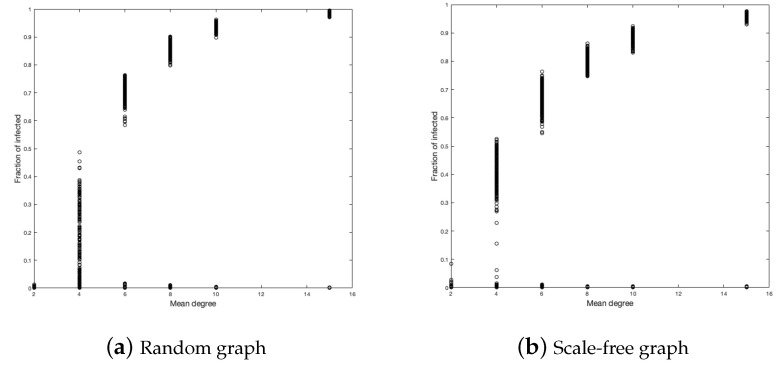
Infected fraction in random and scale-free graph.

The next variable to study is the mean degree of each vertex of the graph. For this purpose, we ran 500 simulations for each graph with a mean of degree equal to 2, 4, 6, 8, 10, and 15. Figure 7 and Figure 8 show the fractions of infected and the duration of the disease.

For the random graph, in the fraction of infected, it is clear that, the larger the average number of contacts, the more infected we will have. This should not be strange because the more contacts, the more likely you are to be infected. The case for d=4 is curious since it presents greater heterogeneity in the simulations.

**Figure 9:**
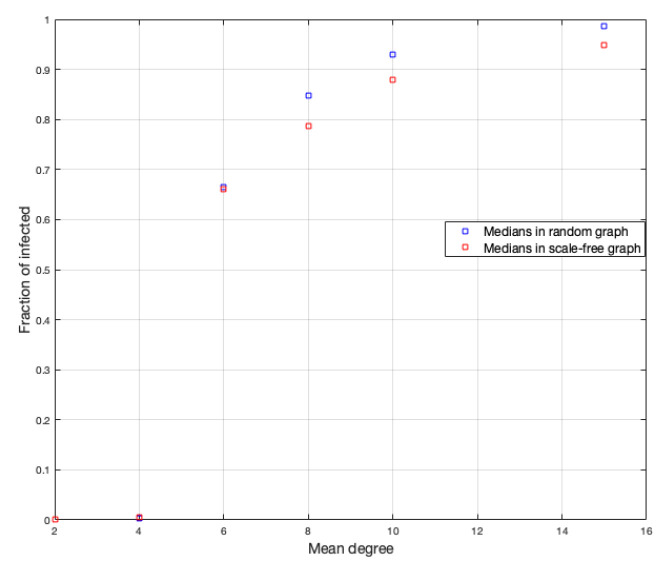
Median for random and scale-free graph in fraction of infected.

In the case of the scale-free graph, something similar happens to the case of the random graph. The case d=4 is also the most heterogeneous, but to a lesser degree than in the case of the random graph. We calculate the medians of each simulation for each *d* to better see the influence from the mean value of the vertices on the infected fraction, as shown in Figure 9. (We choose the median to reduce the bias of extreme data close to zero).

Regarding the duration of the disease, we have in the scale-free graph that, as the average number of neighbors (*d*) increases, the duration of the disease decreases, but not as fast as in the case of the random graph and also the duration is lower in each vertex average with respect to the random graph.

### 4.4. Amount of Relation Variables

Studying the effects of this parameter is important because it is a factor that is not always considered when it comes to modeling the spread of diseases on networks. It is clear that, by changing the amount of variables, the weights assigned to them and consequently the weights assigned to the links of the graph also change. To see this effect, first let’s see how the link weights change when modifying the amount of variables.

**Figure 10:**
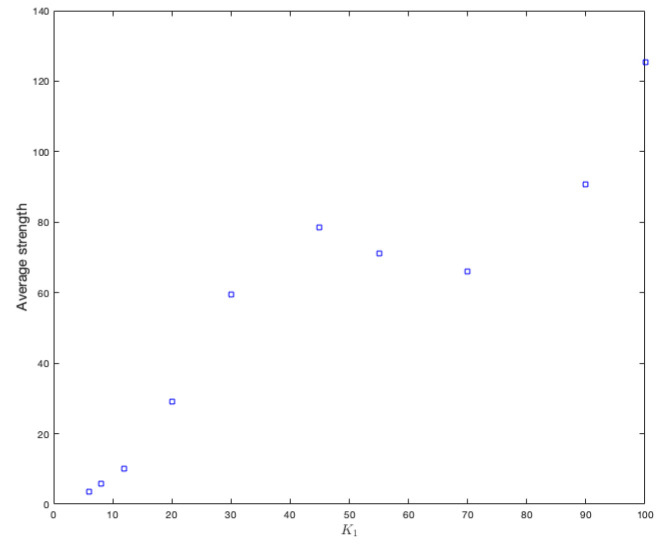
Average strength of vertices for each value of $K_{1}$.

As this assignment is independent from the type of graph, we have only made the changes on a random graph. Our focus was placed on the variation of the average strength that each vertex has, considering 10 different values for $K_{1}$, these are 6,8,12,20,30,45,55,70,90, and 100. To properly observe the effect on the strength of the vertices, we calculate the average strengths of the vertices for each value of $K_{1}$. Figure 10 shows the results. It can be clearly seen that increasing the amount of variables increases the strength of each vertex.

**Figure 11:**
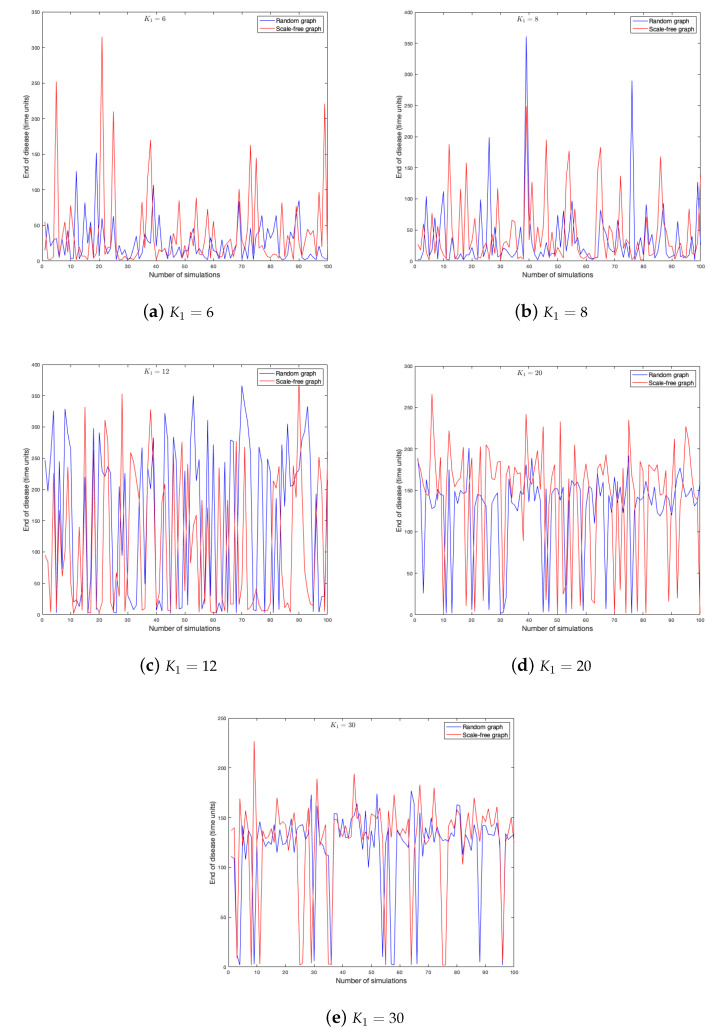
End of disease for different $K_{1}$ in random and scale-free graph.

**Figure 12:**
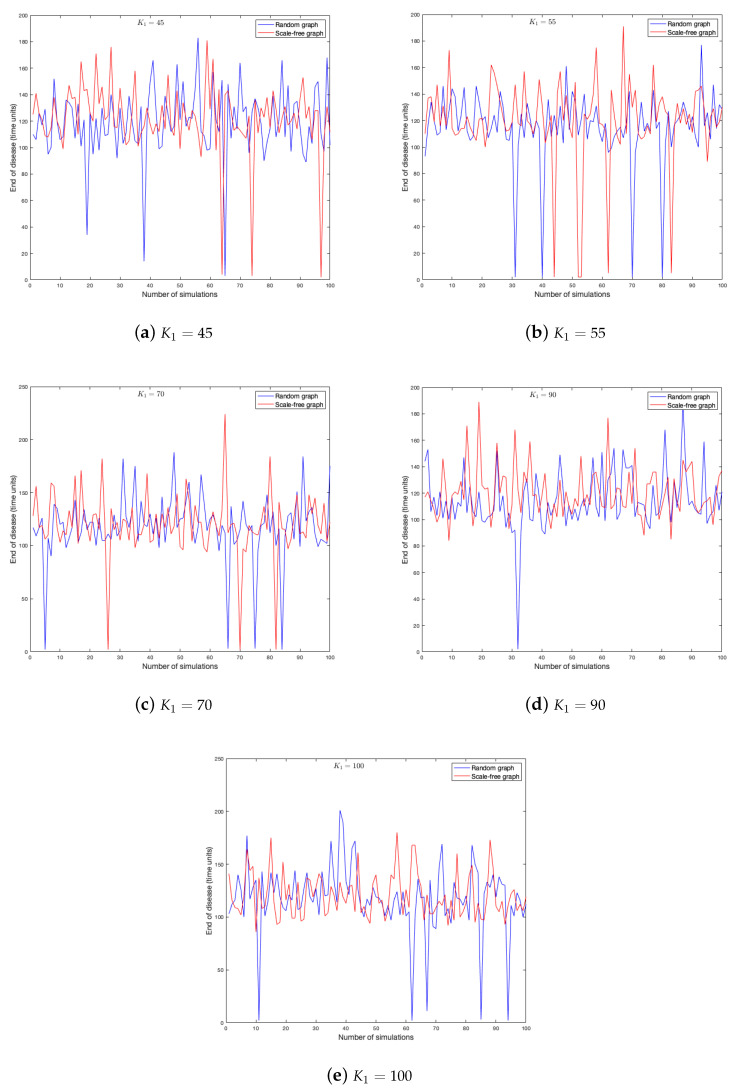
End of disease for different $K_{1}$ in random and scale-free graph.

**Figure 13:**
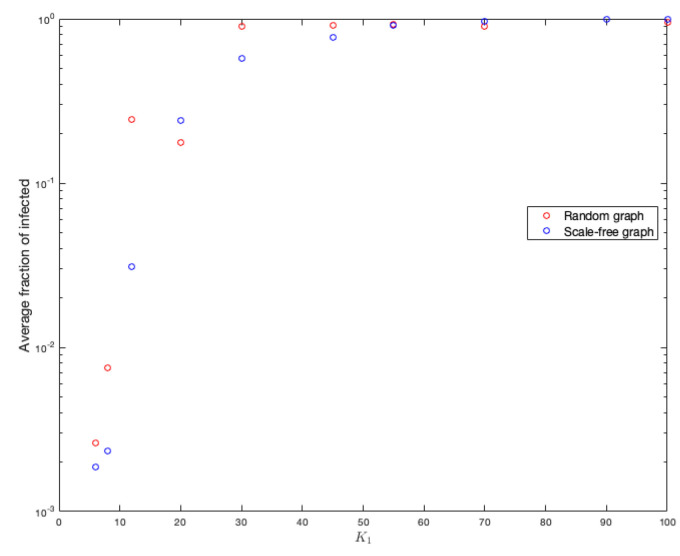
Average infected for the different values of $K_{1}$ in random and scale-free graph.

As the probability of being infected depends on the strength of the neighbors, it is clear that this parameter has effects on the spread of a disease in a graph. One hundred simulations were run on each graph. Figure 11 and Figure 12 show the duration of the disease in random and scale-free graph for each value of $K_{1}$. Figure 13 shows the effects of this parameter on the fraction of infected.

**Figure 14:**
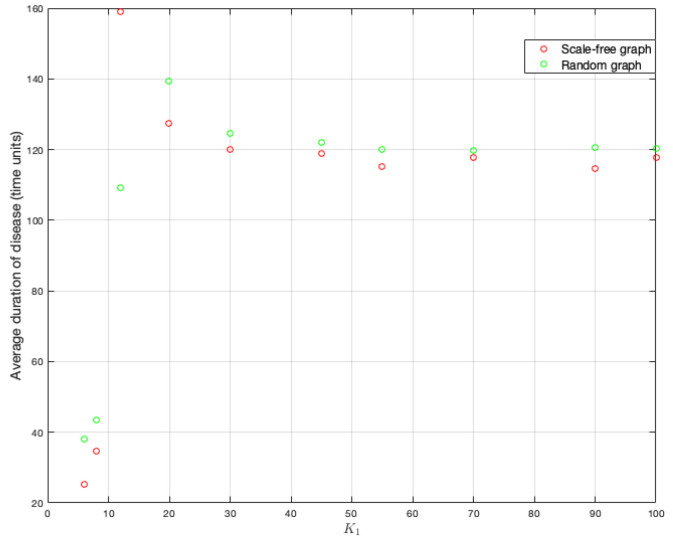
Average end of disease for different $K_{1}$ in random and scale-free graph.

To better understand the figures above, we compute end-of-disease averages for each $K_{1}$ value. It can be observed in Figure 14 that scale-free graph has a shorter duration on average (as it has been verified when studying the effects of other parameters). There is also a tendency to stabilize around 120 time units on average.

On the other hand, in the same way, to better study and see the effects of this parameter on the fraction of infected, the means of each simulation were calculated for each value of $K_{1}$. Figure 13 shows the results.

Notice that the number of infected is slightly higher in random graphs.

### 4.5. The Amount of Classes

**Figure 15:**
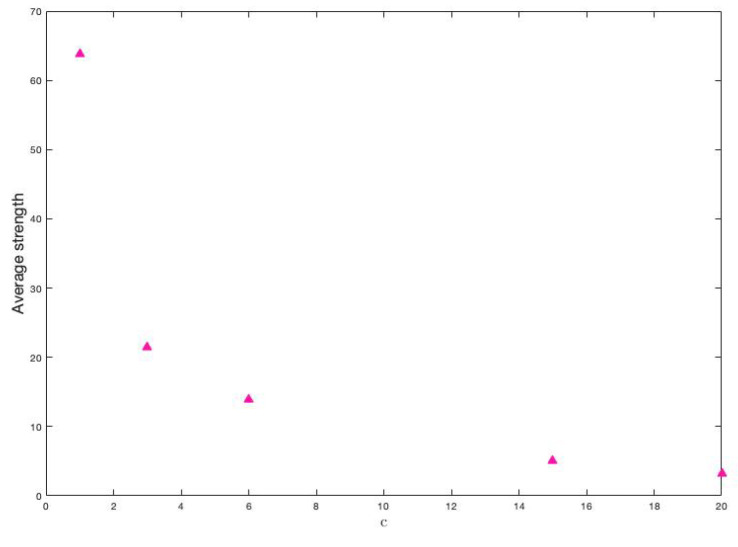
Average strength of vertices for each value of *c*.

The amount of different classes is also a parameter that our proposal considers. Although this is a factor that is determined exclusively by the researcher, we want to study its direct influence on the two characteristics of the disease. Notice that, only by changing the amount of variables, their weights’ assignment change and, consequently, the weights of the links in the graph change as well. The same happens if the amount of classes changes. To see this effect, let’s first see how the weights link change when the amount of classes changes. We have considered the following values for *c*: 1,3,6,15 and 20. The average of these strengths is seen in Figure 15.

It is clear that, if the amount of classes tends to be the number of variables, then the average strength of the vertices decreases. It is also observed that the decrease is possibly exponential.

**Figure 16:**
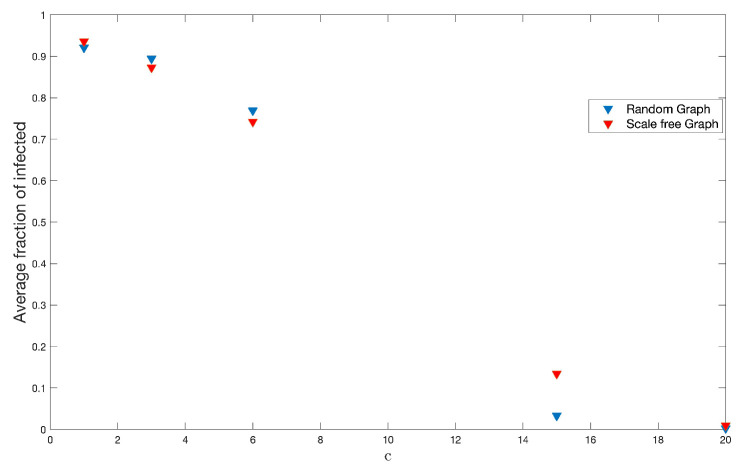
Infected average for the different values of *c* in random and scale-free graph.

**Figure 17:**
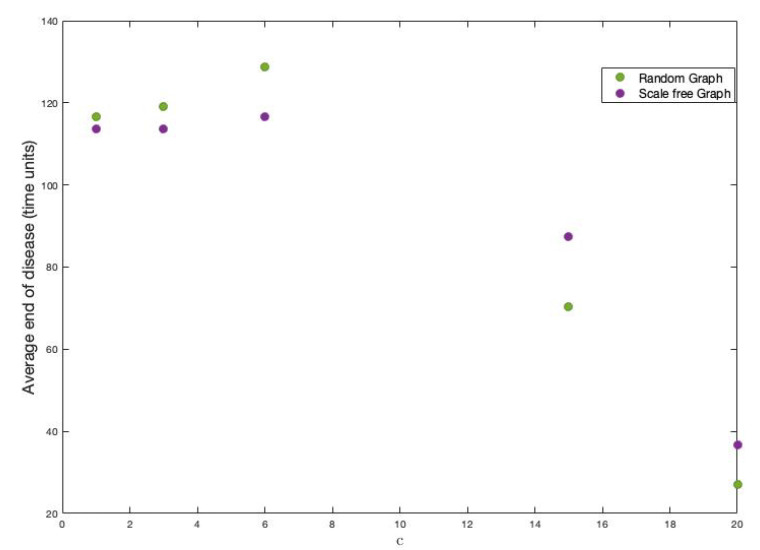
Average end of disease for different *c* in random and scale-free graph.

On the other hand, we have done 200 simulations for each one of these different values of *c* on each type of graph used throughout this work and, with this, to see the effects on the fraction of infected and the end of the disease. Figure 16 and Figure 17 show the results.

## 5. Deterministic Approximation

Despite the previous conclusions, with respect to the different simulations, we will show an approximation of Susceptible, Infected, and Recovered curves through a differential equation system. The idea is to obtain a differential equation system with the parameters that define the graph.

Let I(t)∈[0,N] be the number of infected individuals between two consecutive (discrete) times, i.e.,

(10) $$I\left(t+\Delta t\right)=I\left(t\right)-\delta \Delta tI\left(t\right)+\rho \Delta t\varphi \left(t\right)\left(N-I(t)-R(t)\right),$$

where φ(t) is an estimate of the mean value of strength of infected neighbors for every susceptible individual. In order to get φ(t), we assume that *m* is the neighbors average and that a proportion of these neighbors, we say $\frac{I(t)}{N}$, is infected. On the other hand,

(11) $$\bar{S}=\frac{\sum_{i=1}^{N}S\left(v_{i}\right)}{N}=\frac{\sum_{i=1}^{N}\sum_{v_{j}\in N\left(v_{i}\right)}{\tilde{w}}_{ij}}{N}$$

is the average strength of the graph. Then,

(12) $$\varphi \left(t\right)=\bar{S}\cdot \frac{I(t)}{N}.$$

Replacing the Equation (12) in (10) and as N=S(t)+I(t)+R(t), then

(13) $$I\left(t+\Delta t\right)=I\left(t\right)-\delta \Delta tI\left(t\right)+\rho \Delta t\cdot \bar{S}\frac{I(t)}{N}S\left(t\right).$$

Dividing by Δt and taking the limit as Δt→0, we obtain

(14) $$\dot{I}\left(t\right)=\frac{\rho \cdot \bar{S}}{N}I\left(t\right)S\left(t\right)-\delta I\left(t\right).$$

In the same way, we obtain the equations

(15) $$\begin{array}{cc} \dot{S}\left(t\right) & =-\frac{\rho \cdot \bar{S}}{N}I\left(t\right)S\left(t\right) \end{array}$$

(16) $$\begin{array}{cc} \dot{R}\left(t\right) & =-\delta I(t). \end{array}$$

We can see that the Equations (14)–() are the same as those defined for the SIR model. From there, we can see that $\beta =\frac{\rho \cdot \bar{S}}{N}$.

If the previous deductions are done on a not edge-weighted graph, then we obtain that $\beta =\frac{\rho \cdot m}{N}$. If $\bar{w}$ is the average of weights, then $\bar{S}=\bar{w}\cdot m$. It is clear that, if $\bar{w}=1$, then we are in the case where the graph is not an edge-weighted graph.

**Figure 18:**
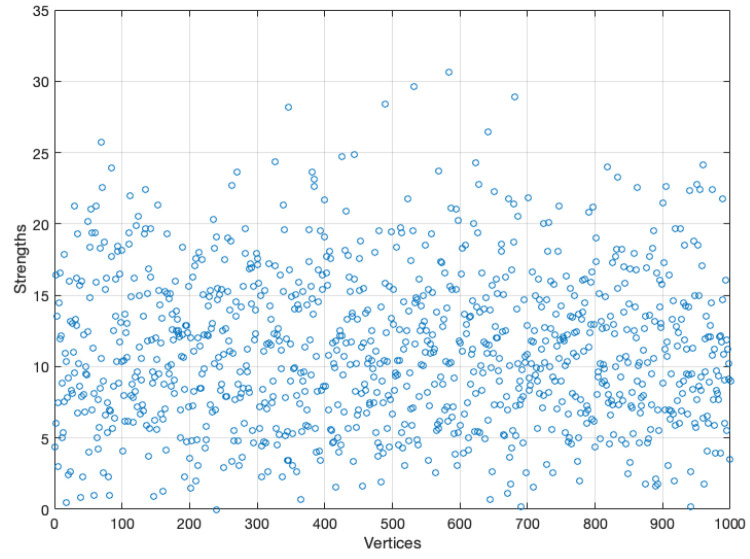
Strength of each vertex.

The above is valid only if the population is mixed, the graph has a fixed contact structure, and all vertices have approximately the same number of neighbors and approximately the same strength. However, the last condition is a stronger condition and certainly; it is not true, even to assume $\bar{S}=\bar{w}\cdot m$ can be a mistake because $\bar{w}$ could be unrepresentative. For instance, in Section 4, the average strengths are not representative in the initial simulation since the strengths are strongly heterogeneous (see Figure 18).

**Figure 19:**
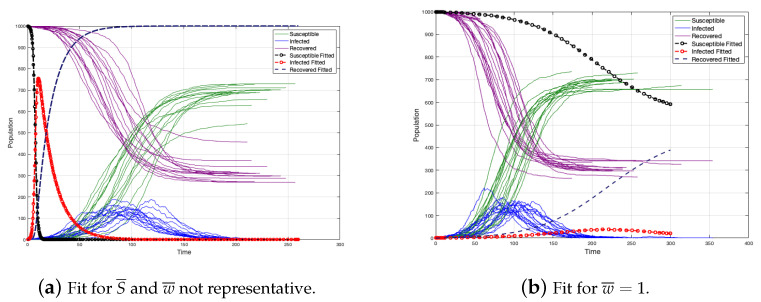
Different fit for $\bar{w}$.

**Figure 20:**
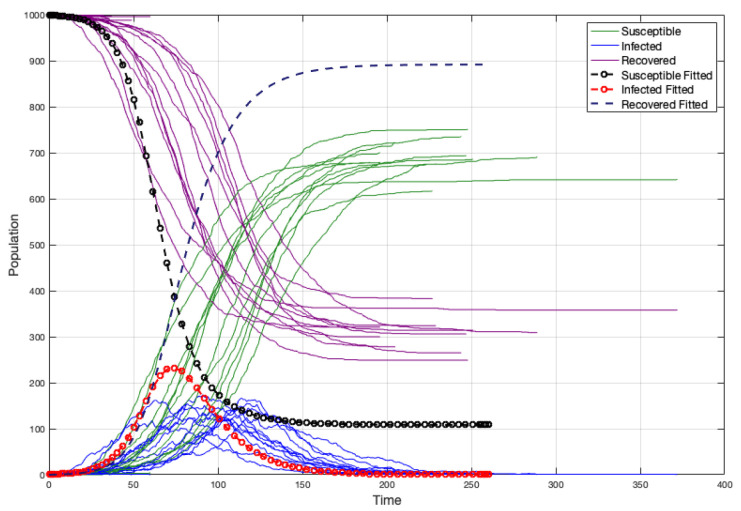
Curves fitted without considering $\bar{S}=\bar{w}\cdot m$.

When the strength and weight average are not representative, there is an overestimation of the infected individuals and the duration of the disease is lower. (see Figure 19a). Moreover, if we consider $\bar{w}=1$ like in the case of the non edge-weighted graph, we have a sub-estimation of the infected individuals and the duration of the disease is upper (see Figure 19b). Certainly, without considering $\bar{S}=\bar{w}\cdot m$, the fit is better, but not really good (because $\bar{S}$ is not a good representative) (see Figure 20).

**Figure 21:**
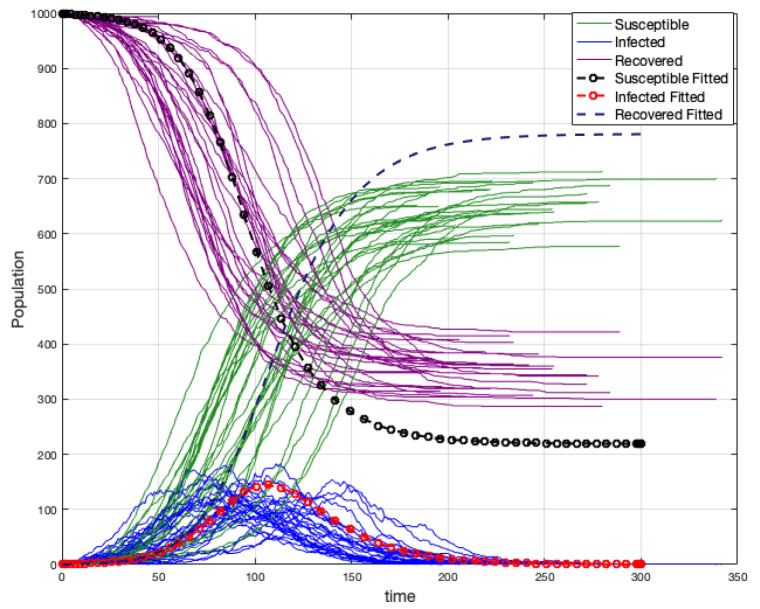
Fitted curves in a random graph with $\psi =\frac{\bar{S}+m}{2}$.

After many simulations (1000), we have noticed that a simple approximation to β is the average between $\bar{S}$ and *m*, in the case where the strength of vertices is not strongly homogeneous (see Figure 21). Thus, if $\psi =\frac{\bar{S}+m}{2}$, we have

(17) $$\begin{array}{cc} \dot{S}\left(t\right) & =-\frac{\rho \psi }{N}I\left(t\right)S\left(t\right) \end{array}$$

(18) $$\begin{array}{cc} \dot{I}\left(t\right) & =\frac{\rho \psi }{N}I\left(t\right)S\left(t\right)-\delta I\left(t\right) \end{array}$$

(19) $$\begin{array}{cc} \dot{R}\left(t\right) & =-\delta I(t). \end{array}$$

A similar problem is treated in [28] in the case of a non edge-weighted graph, where a graph has a heterogeneous distribution degree of vertex (scale-free graph).

## 6. Modeling

**Figure 22:**
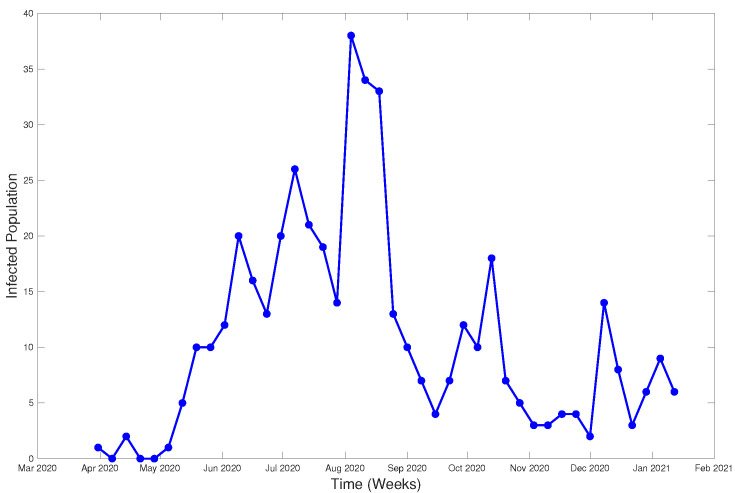
Infected from March 2020 to January 2021, Olmué city (Chili).

The modeled data were obtained from the database of the Epidemiological Surveillance System of the Chilean Ministry of Health, with the approval of the ethics committee of the Faculty of Medicine of the University of Valparaíso (Act No. 15/2020). This system is the official system of the country and allows health management of notifiable diseases, including COVID-19. For the purposes of this study, the database from Olmué city (Valparaíso Region) was used, which included reported cases (positive or negative) and their contacts from 3 March 2020 to 15 January 2021 with *n* = 3866 registered persons. Figure 22 shows the evolution of the infected per week.

**Figure 23:**
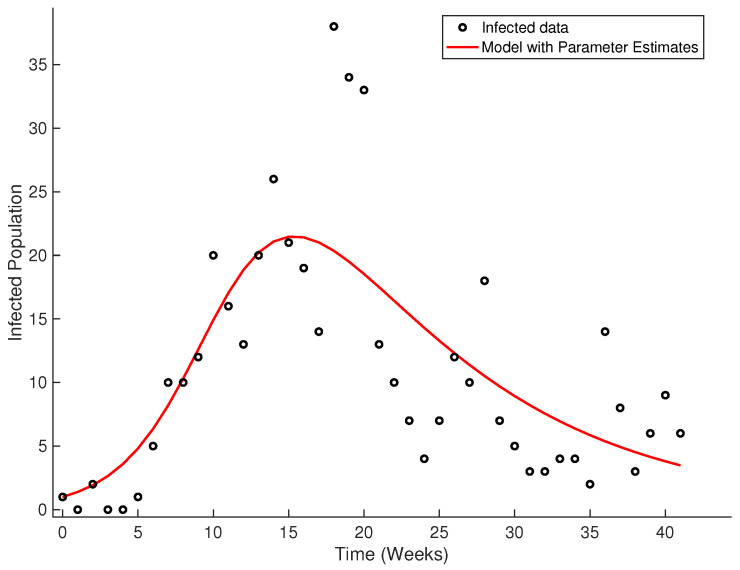
Fitted curve from real data of Olmué city, Chili.

To fit a curve to the data following the SIR model, we used the classic method of least squares. The values for the β and δ parameters are 0.4349 and 0.0937, respectively. Figure 23 shows the fitted curve.

On the other hand, from the total of variables included in the database (K=279), seven of them are relationship variables ($K_{1}=7$). They are: full address ($X_{1}$), the street where the people live ($X_{2}$), town ($X_{3}$), place of work ($X_{4}$), workplace section ($X_{5}$), health facility where they were treated ($X_{6}$), and the region of the country where the test was taken to confirm, or not, the contagion ($X_{7}$).

In our criteria, the hierarchical order of the seven variables in descending form is $X_{1},X_{2},X_{3},X_{4},X_{5},X_{6},X_{7}$. Moreover, we consider that the variables $X_{1},X_{2}$ and $X_{3}$ have the same weight. In the same way, we also consider the variables $X_{4}$ and $X_{5}$ with equal weight. Hence, $A_{1}=\left\{X_{1},X_{2},X_{3}\right\}$, $A_{2}=\left\{X_{4},X_{5}\right\}$, $A_{3}=\left\{X_{6}\right\}$ and $A_{4}=\left\{X_{7}\right\}$ are the different classes that are defined by the different weights. Hence, by Definition 10,

$$p_{1}=\frac{3}{7},\;p_{2}=\frac{2}{7},\;p_{3}=\frac{1}{7},\;p_{4}=\frac{1}{7}.$$

**Figure 24:**
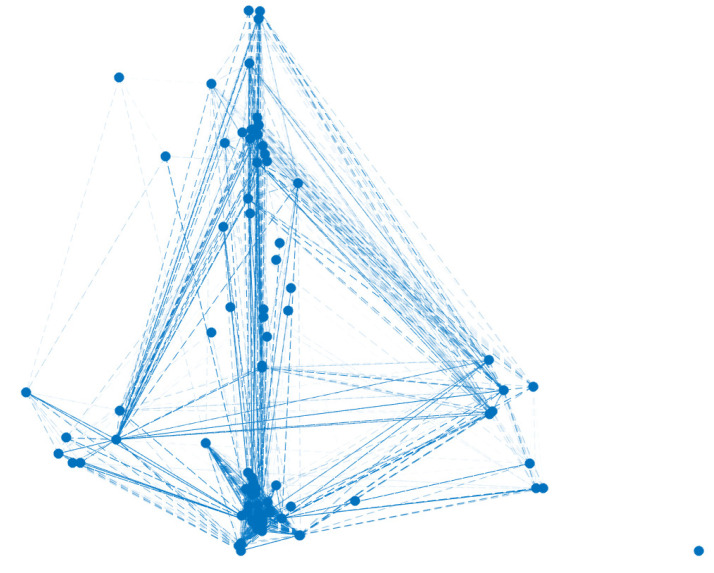
Graph obtained from database of Olmué city, Chili, with 3866 vertices and 6,841,470 edges.

Figure 24 shows the obtained graph.

**Figure 25:**
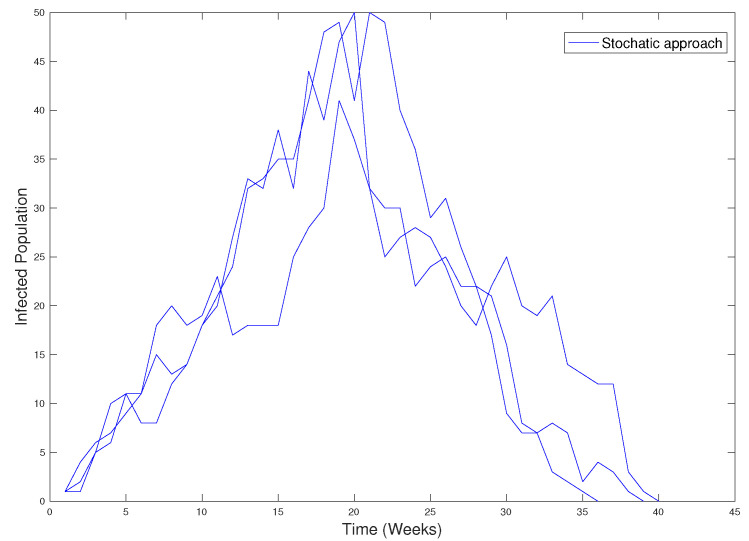
Simulations on graph obtained in Figure 24.

We have run some simulations of the spread of the disease on the graph obtained from the data base of the city of Olmué, considering the recovery rate $\delta =\frac{1}{14}$ and ρ=0.00125. The results are in Figure 25.

**Figure 26:**
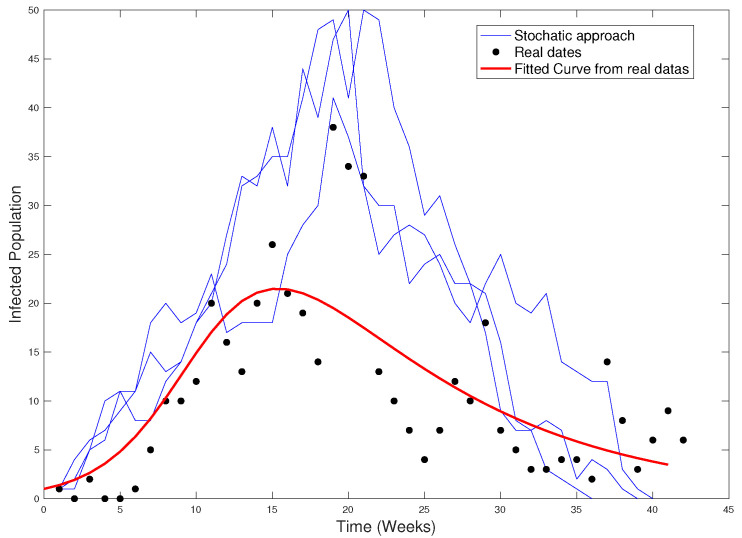
Real data (black), stochastic approach (blue), and fitted curve (red).

Figure 26 shows the real data, the fitted curve, and the stochastic approach.

Notice that, in this case, it is not possible a deterministic approximation like in Section 5 because the average strength is not a good representative.

## 7. Discussion

Our proposal confirms the possibility of obtaining an edge-weighted graph from individuals registered in a database. The decision to incorporate weights on its edges is an attempt to quantify the ties between individuals. An important assumption for the quantification of these weights is to recognize a certain value in each variable or a certain hierarchical order between them, that is, to identify which variable (s) is (are) more important than others and defining an order. In this way, it is possible to define the so-called classes of variables (that is, each class is defined by variables that have the same value or defined hierarchical order), since it is possible that two or more variables have the same value or hierarchical order. This is similar to the stated by Enright & Raymond, who hold that the weighting of the edges is relevant for a more comprehensive understanding of the disease dissemination processes, without losing information provided by the network [12]. In particular, this research provides an approach that relates population groups and discrimination variables of greater or lesser force for the spread of diseases, which allows for understanding the level of interaction between people. This, for Keeling & Eames, is paramount for understanding infectious diseases [29].

Consequently, for the case study (Olmué city), the model was adjusted to the spread of the disease, both in the number of people with COVID-19 and the duration of the disease. This shows that both the theoretical and practical development are useful from a public health perspective. This is relevant due to the weighting of the relationship variables.

Likewise, our proposal of weighted graphs obtained from a database would be useful for health organizations and scientific teams as a tool for the modeling of infectious diseases through a database in order to establish priority variables in the understanding of these health events, in public health surveillance, and in the establishment of measures for the prevention of infections with greater certainty [16,30]. For this reason, it is important to have standardized databases that allow the evolution of health problems to be analyzed with a greater degree of complexity [31]. As an example, the COVID-19 database could contain other relationship variables that would allow us to know the interaction between people who are susceptible to get infected or who are sick with SARS-COV2. In summary, we believe it is relevant to continue with data collection efforts in a systematic way by scientific and governmental organizations [32].

Our work coincides with the stated by Keeling & Eames regarding the integration of complex networks and their limitations in relation to having an adequate volume of data to represent infectious diseases [29]. However, the limitations that the authors mention are diminishing, since, for the management of infectious diseases, most of the countries make greater efforts in the systematization of information [33], as well as the COVID-19 pandemic has reaffirmed these actions.

After performing simulations to study each effect of the parameters described, we can point out that, regardless of the parameter that is intervened, the durations of the diseases are shorter on scale-free graphs than on random ones. This may be due to the heterogeneity and recognition of infectious disease propagators provided by free-scale graphs [29]. For its part, the number or fraction of infected is always greater on random graphs than on scale-free graphs. The only parameter in which this was not evident is for the purely biological disease factor ρ. This would be generated because the random network does not discriminate the level of targeted contacts and gives the chance of contagion [29]. An interesting fact is the case where we vary the amount of classes. If the class number tends to be the same as the amount of variables, the average strength increases, and this means that the fraction of infected also increases.

We believe that it would be very interesting, and complementary to our proposal, to carry out simulations on other types of graphs, for example graphs of small worlds and their different variations. It is very likely that the spread of diseases on these graphs differ from the results obtained on the graphs used in this work. Studying the effects of other parameters on the disease would also be beneficial. For example, we believe that an interesting factor would be the number of initial infected and what it happens if they are neighbors or to what extent the disease spreads more with respect to the distance between them. In this same sense, varying the strength of the infected vertex would also give us new information.

The deterministic approach is undoubtedly a useful tool, but it lacks being generic, in the sense that in order to make use of it, some fairly strong assumptions are necessary, they are the homogeneity in the number of neighbors and the average strength for each vertex. Therefore, in scale-free graphs, we cannot use this approximation since the average of neighbors by vertices is not representative from the reality of the graph.

The importance of having accurate models able to explain the spread of epidemics and resemble reality lies in understanding it for the development of effective defense strategies against contagious diseases. Current demands drive progress in this field, which will undoubtedly continue to grow. It is important to mention that these types of contributions are only one dimension in the understanding of epidemic events. In this sense, for a holistic approach, it is necessary to include analyses of different disciplines such as the social, biological, and health sciences and, above all, the meanings that populations give to pandemics. 

## Figures and Tables

**Table 1:** Database D.

Person	City	Workplace	E. C. Activity	Address	Sm.	Dri.	Gen.	M. S	Age
1	A	Workplace 1	Theater	y	Y	Y	F	IC	35
2	A	Workplace 3	Cinema	y	Y	Y	M	IC	35
3	B	School B	Football	z	N	N	F	S	10
4	B	Workplace 1	Photography	x	N	N	F	M	48
5	A	Workplace 5	Does not have	u	Y	N	F	W	65
6	A	Workplace 4	Does not have	v	Y	Y	M	IC	27
7	B	Workplace 2	Does not have	x	Y	N	M	M	46
8	A	University 1	Photography	v	N	N	M	IC	29
9	A	University 2	Does not have	w	Y	Y	M	IC	19
10	B	School B	Karate	x	N	N	M	S	10
11	A	Workplace 4	Ping-pong	r	Y	Y	F	M	54
12	A	School A	Football	s	N	N	M	S	8
13	A	Workplace 5	Dance	r	Y	Y	F	M	57
14	B	School A	Handball	q	N	N	M	S	11
15	A	University 1	Does not have	t	N	N	F	S	25
16	A	Workplace 7	Singing	p	Y	Y	F	S	60
17	A	Workplace 8	Music	k	N	Y	F	S	28
18	A	Workplace 3	Does not have	d	N	N	M	S	47
19	B	School A	Music	g	N	N	F	S	8
20	A	Workplace 6	Does not have	h	Y	Y	M	S	30

## Data Availability

The data presented in this study were available after being requested by research project COVID-ANID to the Chilean Ministry of Health. The data are not publicly available due to legal restrictions.
